# Individual and population level impact of chronic conditions on functional disability in older adults

**DOI:** 10.1371/journal.pone.0229160

**Published:** 2020-02-20

**Authors:** Parminder Raina, Anne Gilsing, Alexandra J. Mayhew, Nazmul Sohel, Edwin van den Heuvel, Lauren E. Griffith

**Affiliations:** 1 Department of Health Research Methods, Evidence, and Impact, McMaster University, Hamilton, Ontario, Canada; 2 Labarge Centre for Mobility in Aging, McMaster University, Hamilton, Ontario, Canada; 3 McMaster Institute for Research on Aging, McMaster University, Hamilton, Ontario, Canada; 4 Department of Mathematics & Computer Science, Eindhoven University of Technology, Eindhoven, The Netherlands; Universita degli Studi di Napoli Federico II, ITALY

## Abstract

**Background:**

It is unknown if the relationship between multimorbidity and disability differs by combinations of chronic conditions. The objective of our study was to elucidate how joint effect of different combinations of chronic conditions impact the five year risk of functional disability at the population level.

**Methods:**

Participants ≥65 years from the Canadian Study of Health and Aging were assessed for functional disability measured using activities of daily living (ADL) and instrumental ADL (IADL), and the presence of conditions in five disease domains; cardiometabolic, neurological, sensory, musculoskeletal, and respiratory. Logistic regression was used to assess the relationship between each disease domain and incident ADL and IADL measured at five years of follow up and population attributable risk (PAR) was modeled for diseases domains that were significantly associated with disability. Results were stratified by sex and age (65–74 years, ≥75 years).

**Results:**

There were 6272 participants free of ADL disability and 4571 participants free from IADL disability at baseline. For incident ADL, the greatest PAR values were 21.3 (9.8–32.8) for the cardiometabolic domain in males 65–74 years, 22.7 (4.7–40.8) for the musculoskeletal domain for females aged 65–74 years, and 11.2 (2.8–19.7) for the musculoskeletal domain in males ≥75 years. The PAR for the musculoskeletal, sensory, and neurological domains were similar in females ≥75 years(9.3–9.9). PAR values were lower but followed similar patterns for IADL disability.

**Conclusion:**

The chronic disease domains which most strongly predicted incident ADLs and IADLs did not account for the greatest amount of disability at the population level.

## Introduction

Increasing life expectancy and declining fertility rates have led to an ageing population worldwide [1]. In 2015, the global population of people aged 60 years or older was 900 million and it is expected to rise to two billion by 2050 [2]. The prevalence of chronic conditions increases with age as does the risk of having two or more chronic conditions which is referred to as multimorbidity [1,3]. Between 34% and 61% of older adults have multimorbidity depending on the definition used and population studied. [4] Multimorbidity is associated with increased risk of death, disability, and impaired function [5,6]. There is evidence that certain combinations of chronic conditions may increase the risk of disability beyond what would be expected based on the individual effect of each condition on disability alone [7–9]. However, many of these studies failed to find differences between specific combinations of conditions, in part due to the small number of participants with each combination of chronic disease conditions. These studies relied on regression modeling which provides information about the association between chronic conditions and health on an individual level, but did not provide insight about the relationship in the population. Consequently, it is unknown what impact the joint effect of different chronic disease conditions have on disability at the population level.

From a public health perspective, it is essential to determine the burden of functional disability in the population attributable to different chronic condition combinations. Public health interventions targeting conditions that modestly increase the risk of disability, but are highly prevalent in the population (e.g. heart disease), may have a larger impact on the incidence of functional decline in the population compared to conditions strongly associated with disability but that are less prevalent in the population (e.g. stroke, Parkinson’s disease, hip fractures) [10]. The few studies that have reported the population impact of multimorbidity are limited by the use of cross-sectional data, hampering causal inference [11–13]. Associations between functional disability and some of the conditions reported in these publications, may have suffered from reverse causality [14]. As well, previous investigations have mostly focused on single conditions and seldom examined the impact of combinations of chronic conditions. Given that many of the common co-existing chronic conditions share risk factors [15], knowledge about which combinations of chronic conditions have the greatest impact on functional disability in the population may help identify efficient targets for complex, multicomponent, public health interventions.

The objective of our study was to elucidate how the joint effect of different combinations of chronic conditions impacts the five year risk of functional disability in adults over the age of 65 years at the population level.

## Methods

### Study design and population

This was a secondary data analyses of data from the Canadian Study of Health and Aging (CSHA) which was approved as project number 01–121 by the McMaster University and Hamilton Health Sciences Corporation Research Ethics Board. The CSHA is a national, population-based study of cognitive impairment and other aspects of health in Canadian adults aged ≥65 years [16]. In the first wave of the CSHA in 1991–1992, face-to face interviews were conducted with 9008 individuals living in the community and 1255 individuals living in institutions. Follow up interviews were conducted five years later (1996–1997). These participants were recruited from 39 urban centers and surrounding rural areas in the ten Canadian provinces. The present analysis is restricted to the 9008 participants from the community dwelling sample. Of these participants, 8439 (93·7%) had complete ADL, IADL and chronic disease data at baseline. There were 575 participants who did not attend follow up that were excluded from analyses. Individuals with an ADL disability at baseline (n = 1079) were excluded from the ADL analyses, whereas subjects with an IADL disability (n = 2621) were excluded from the IADL analyses. Participants with incomplete disability data (ADL n = 120, IADL n = 199), who were either decreased, unable to complete the interview, or who were cognitively impaired without proxy data (ADL n = 393, IADL n = 473) were excluded. Overall, 6272 participants were available for the ADL analyses and 4571 for the IADL analyses.

### Functional disability

At baseline and follow up, functional status was measured by trained staff using the Older Americans Resources and Services (OARS) Multidimensional Functional Assessment Questionnaire [17]. The questionnaire includes seven items assessing basic ADLs (eating, dressing, putting on clothes, walking, getting to bed, bathing and toileting) and seven assessing IADLs (telephone use, travel, shopping, meal preparation, housework, taking own medicine, and handling personal finances) ADL and IADL disability were defined as needing help with, or an inability to perform one or more of the ADL or IADL activities respectively. When subjects were deceased, unable to complete the interview or found to be cognitively impaired, follow-up reporting of disability was completed by a proxy who knew the subject well.

### Chronic conditions

Based on the literature, five body system specific disease domains encompassing several chronic conditions each were identified a priori as risk factors for functional disability: 1) cardiometabolic, 2) neurological, 3) sensory, 4) musculoskeletal, and 5) respiratory conditions. [18] Cardiometabolic conditions included heart problems (history of hardening of the arteries, heart troubles or other blood diseases) and diabetes. Neurological conditions included stroke, Parkinson’s disease, and cognitive impairment. Cognitive status was assessed with the Modified Mini-Mental State examination; a score ≤77 was used to define cognitive impairment [19]. Sensory conditions included vision problems and hearing problems measured using self-reported presence or absence of eye trouble not resolved by glasses and ear trouble. Musculoskeletal conditions included any kind of foot or ankle problem, any kind of fracture, and arthritis. Respiratory problems included asthma, pneumonia, tuberculosis, emphysema, bronchitis and breathing problems (asked as one item in the questionnaire). The conditions were self-reported at baseline, and caregivers provided proxy information when participants could not answer for themselves. Participants were considered to have a disease within a domain if they had at least one chronic condition belonging to that domain.

### Sociodemographic factors

Sociodemographic factors included age, gender, marital status, level of education and living situation. Age was categorized into two groups: 65–74 years and ≥75 years. Marital status was categorized into married, never married and widowed/separated/divorced. Level of education was divided into two categories: zero to nine years and ten or more years. Living situation was categorized as living alone or living with someone.

### Statistical analyses

To investigate the contribution of individual conditions to the selected disease domains, prevalence of disability was calculated, stratified by the disease domains and their individual components. The association between disease status at baseline and incidence of disability was examined using logistic regression models. Analyses were stratified by age (65–74, 75+ years) and sex. Models were adjusted for marital status, level of education, living situation, and the other disease domains.

Model-based, adjusted estimations of population attributable risk (PAR) were computed to explore the population impact of disease domains on incident functional disability [20,21]. Analyses were restricted to disease-disability associations with p-values <0.05 from the logistic regression models described above. The PAR and 95% confidence intervals were estimated based on a series of unconditional multivariable logistic regression models using interactive risk attributable program software (US National Cancer Institute, 2002). PARs were calculated for individual conditions and combinations of conditions. PARs were ranked and compared qualitatively.

## Results

### Participant characteristics

In the ADL sample, the mean age of participants was 75·8 ± 6·6 years, 44·3% of participants were men and 55·9% were married. The mean age in the IADL sample was 74·5 ± 6·0, 47·5% participants were male, and 59·7% were married. About a third of participants lived alone, and the mean number of years of formal education was ten years in both samples.

### Chronic disease domains prevalence and disability incidence

In the ADL sample, 85·5% of participants had at least one chronic disease domain compared to 82·2% of participants in the IADL sample (Table 1). The majority of participants reported having chronic conditions in two or more domains. For both samples, the most prevalent disease domain was musculoskeletal (ADL 64·1%, IADL 60.9%) and the least prevalent was neurological (ADL 14·8%, IADL 11·4%). For all domains, the prevalence was higher in older (≥75 years) versus younger (65–74 years) participants. The five year incidence of functional disability in ADL was 27·1% and the incidence of IADL disability was 40.2% with higher incidence in participants ≥75 years compared to those aged 65–74 years.

**Table 1:** Distribution of disease clusters and incident disability in the Canadian Survey on Health Aging, stratified by age.

	Activities of daily living sample			Instrumental activities of daily living sample		
	Total population	65–74 years	75+ years	Total population	65–74 years	75+ years
	n = 6272	n = 2884	n = 3388	n = 4571	n = 2439	n = 2132
	N (%)	N (%)	N (%)	N (%)	N (%)	N (%)
**Males**	2776 (44.3)	1350 (46.8)	1426 (42.1)	2170 (47.5)	1188 (48.7)	982 (46.1)
***Disease clusters***						
no clusters	908 (14.5)	563 (19.5)	345 (10.2)	813 (17.8)	532 (21.8)	281 (13.2)
one cluster	1931 (30.8)	1025 (35.5)	906 (26.7)	1568 (34.3)	915 (37.5)	653 (30.6)
two+ clusters	3433 (54.7)	1296 (44.9)	2137 (63.1)	2190 (47.9)	992 (40.7)	1198 (56.2)
**Disease domain**						
cardiometabolic	2095 (33.4)	887 (30.8)	1208 (35.7)	1318 (28.8)	676 (27.7)	642 (30.1)
musculoskeletal	4018 (64.1)	1766 (61.2)	2252 (66.5)	2784 (60.9)	1439 (59)	1345 (63.1)
sensory	2752 (43.9)	955 (33.1)	1797 (53)	1787 (39.1)	733 (30.9)	1034 (48.5)
respiratory	1006 (16)	426 (14.8)	580 (17.1)	609 (13.3)	312 (12.8)	297 (13.9)
neurological	931 (14.8)	247 (8.6)	684 (20.2)	520 (11.4)	180 (7.4)	340 (16)
**Incident disability (5 year)**						
ADL	1699 (27.1)	386 (13.4)	1313 (38.8)			
IADL				1837 (40.2)	712 (29.2)	1125 (52.8)

Though the prevalence of each chronic condition was higher in the ADL sample compared to the IADL sample, the percentage of people that developed an IADL disability in five years was higher for each chronic condition in comparison to those that developed ADL disability (Table 2). For each disease domain, between 30.0% and 46.0% of participants with a chronic condition developed an ADL disability and between 43.9% and 61.7% developed an IADL disability within five years.

**Table 2:** Prevalence of activities of daily living and instrumental activities of daily living limitations at five years of follow up for participants with chronic diseases at baseline.

	Activities of daily living sample			Instrumental activities of daily living sample		
	All n = 6272		Disability	All n = 4571		Disability
	Number of participants at baseline with disease	Prevalence of participants at baseline with disease	Prevalence of ADL limitations at 5 years follow up for participants with chronic disease at baseline	Number of participants at baseline with disease	Prevalence of participants at baseline with disease	Prevalence of IADL limitations at 5 years follow up for participants with chronic disease at baseline
**Cardiometabolic**	2095	33.7%	33.4%	1318	28.8%	49.4%
heart disease	1756	28.0%	33.7%	11077	23.6%	50.0%
diabetes	570	9.1%	37.2%	373	8.2%	52.6%
**Musculoskeletal**	4018	64.1%	30.3%	2787	60.9%	43.9%
arthritis	3413	54.4%	30.2%	2345	51.3%	43.5%
foot problems	1790	28.5%	36.0%	1153	25.2%	50.0%
fractures	339	5.4%	32.7%	222	4.8%	46.8%
**Sensory**	2752	43.9%	33.9%	1787	39.1%	48.1%
vision problems	1673	26.7%	37.7%	1014	22.2%	51.0%
hearing problems	1655	26.4%	32.9%	1078	23.6%	47.9%
**Respiratory**^a^	1006	16.0%	34.6%	609	13.3%	52.9%
**Neurological**	931	14.8%	46.1%	520	11.4%	61.7%
stroke	197	3.1%	41.1%	116	2.5%	60.3%
cognitive impairment	721	11.5%	47.6%	395	8.6%	63.0%
Parkinson’s disease	64	1.0%	42.2%	33	0.7%	63.6%

^a^ respiratory conditions were included as one items in the questionnaire and included asthma, pneumonia, tuberculosis, emphysema, bronchitis and breathing problems

### Association between disease domains and disability

Table 3 shows the association between disease domains and ADL and IADL disability by age and sex. Cardiometabolic conditions were associated with 1·94 (1·38–2·73) greater odds of ADL disability risk in males and 1·88 (1·39–2·55) in females aged 65–74 years. These effects attenuated to a 27% increase in risk in those ≥75 years. In all age and sex groups, muscoskeletal conditions were associated with an increased risk of ADL disability (OR ranging from 1·26–1·51). Sensory conditions were only associated with an increased risk in ADL disability in participants ≥75 years (males: OR 1·34 (1·06–1·68); females: 1·38 (1·15–1·67). Respiratory conditions increased the risk of ADL disability in males and females <75 years (OR 1·56 (1·05–2·32) and 1·93 (1·32–2·82), respectively). This effect was attenuated and was no longer significant in participants ≥75 years. Neurological conditions were associated with a more than 90% increased risk of ADL disability across strata of age and sex. The disease domains had similar effects on the five year incidence of IADL disability both in terms magnitude and age differences (Table 3). Exceptions are sensory conditions, which were also associated with an increased risk of IADL disability in males aged 65–74 years (OR 1·47 (1·12–1·93) and respiratory conditions, which increased the risk of IADL in males ≥75 years by almost 50%.

**Table 3:** Odds of activities of daily living disability and instrumental activities of daily living disability for participants with at least one condition in chronic disease domain after five years of follow up, stratified by age and sex^a^.

	Males				Females			
	Number of participants with disease domain	Number of participants with ADL disability	Odds (95% CI) of ADL disability for participants with at least one condition in chronic disease domain^b^		Number of participants with disease domain	Number of participants with ADL disability	Odds (95% CI) of ADL disability for participants with at least one condition in chronic disease domain^b^	
	**Activities of daily living disability**							
**65–74 years**								
cardiometabolic	441	83	1.94	(1.38–2.73)	446	97	1.88	(1.39–2.55)
musculoskeletal	711	105	1.29	(0.91–1.84)	1055	173	1.51	(1.06–2.15)
sensory	465	74	1.36	(0.97–1.92)	490	85	1.11	(0.82–1.52)
respiratory	233	45	1.56	(1.05–2.32)	193	49	1.93	(1.32–2.82)
neurological	141	31	1.89	(1.20–2.98)	106	31	2.23	(1.39–3.57)
**75+ years**								
cardiometabolic	522	204	1.27	(1.01–1.61)	686	322	1.27	(1.05–1.55)
musculoskeletal	832	317	1.36	(1.08–1.71)	1420	621	1.26	(1.02–1.56)
sensory	738	285	1.34	(1.06–1.68)	1059	489	1.38	(1.15–1.67)
respiratory	296	121	1.28	(0.98–1.68)	284	133	1.12	(0.86–1.46)
neurological	348	165	1.94	(1.50–2.52)	336	202	2.55	(1.99–3.28)
	**Instrumental activities of daily living disability**							
**65–74 years**								
cardiometabolic	369	151	1.78	(1.36–2.34)	307	125	1.91	(1.45–2.53)
musculoskeletal	617	215	1.28	(0.98–1.67)	822	261	1.48	(1.12–1.95)
sensory	388	147	1.47	(1.12–1.93)	365	119	1.10	(0.83–1.45)
respiratory	184	84	1.91	(1.37–2.67)	128	57	1.90	(1.29–2.80)
neurological	114	55	2.07	(1.37–3.11)	66	31	2.05	(1.21–3.46)
**75+ years**								
cardiometabolic	326	191	1.45	(1.10–1.91)	316	184	1.22	(0.93–1.60)
musculoskeletal	555	304	1.16	(0.89–1.51)	790	442	1.33	(1.02–1.72)
sensory	475	272	1.41	(1.09–1.84)	559	322	1.32	(1.04–1.68)
respiratory	170	104	1.49	(1.06–2.11)	127	77	1.27	(0.86–1.87)
neurological	196	132	2.16	(1.54–3.04)	144	103	2.39	(1.61–3.55)

^a^ All analyses adjusted for sex, level of education, marital status and living situation. Models also adjusted for other disease domains.

^b^ Disease clusters: OR for having versus not having at least 1 condition in the cluster

### Population attributable risk (PAR) analyses

When looking at single disease domains, cardiometabolic conditions were the main driver of ADL (21·0%) and IADL disability (12·7%) in younger males (Tables 4 and 5). Musculoskeletal conditions had the largest PAR for ADL (23.0%) and IADL (17·2%) disability in younger females. Musculoskeletal and sensory conditions were drivers of disability in participants 75+ years. When exploring joint effect of two domains, combinations that included the cardiometabolic domain had the largest PAR for ADL and IADL disability in males (65–74 years: PAR range 27·6–31·2; 75+ years: PAR range 17·6–21·0). Combinations that included the musculoskeletal domain were driving the PAR for both types of disability in females (65–74 years: PAR range 25·3–36·7; 75+ years: PAR range 19·0–28·1). In older males and females, combinations including neurological conditions were associated with the highest PARs. The PARs for combinations of three domains were higher but identified similar drivers as combinations of two domains.

**Table 4:** Population attributable risk for activities of daily living disability by disease cluster after five years of follow up, stratified by age and sex^a^^b^.

	Age 65–74				Age 75+			
	Males		Females		Males		Females	
	PAR	95% CI	PAR	95% CI	PAR	95% CI	PAR	95% CI
***1 domain***								
cardiometabolic	**21.3**	(9.8–32.8)	17.5	(8.7–26.4)	5.7	(0.2–11.2)	4.8	(0.9–8.7)
musculoskeletal	12.2	(-4.3–28.8)	**22.7**	(4.7–40.8)	**11.2**	(2.8–19.7)	**9.4**	(1.0–17.8)
sensory					9.4	(2.0–16.9)	**9.9**	(4.2–15.5)
respiratory	8.3	(0.2–16.3)	8.8	(3.0–14.5)				
neurological	7.5	(1.2–13.7)	6.4	(1.9–10.9)	10.8	(6.4–15.1)	**9.3**	(6.7–11.9)
***2 domains***								
card/musc	**31.2**	(15.7–46.8)	**36.7**	(20.2–53.2)	16.6	(7.1–26.1)	14.0	(5.3–22.8)
card/sens	**29.7**	(16.3–43.0)	20.2	(9.2–31.2)	14.8	(6.3–23.4)	14.5	(8.1–20.9)
card/respiratory	**28.0**	(16.0–39.9)	25.0	(15.7–34.3)	8.9	(2.6–15.3)	5.7	(1.5–9.9)
card/neurological	27.6	(16.1–39.1)	23.3	(14.2–32.4)	16.3	(9.5–23.1)	14.1	(9.4–18.8)
musc/sens	21.3	(4.0–38.5)	25.3	(6.9–43.6)	20.2	(10.2–30.1)	**18.9**	(9.7–28.2)
musc/respiratory	19.6	(3.0–36.1)	**30.0**	(12.7–47.2)	14.4	(5.6–23.2)	10.3	(1.8–18.8)
musc/neurological	18.9	(2.4–35.4)	**28.0**	(10.8–45.3)	**21.6**	(12.5–30.7)	**18.7**	(9.9–27.4)
sens/respiratory	17.7	(5.5–29.9)	11.7	(2.1–21.4)	12.7	(4.7–20.7)	10.8	(4.8–16.7)
sens/neurological	19.3	(5.6–33.0)	11.4	(-0.3–23.0)	**28.4**	(17.9–38.8)	**29.2**	(20.9–37.4)
respiratory/neurological	17.5	(6.8–28.2)	17.5	(9.8–25.3)	**20.8**	(13.2–28.5)	16.5	(11.4–21.5)
***3 domains***								
card/musc/sens	**42.5**	(27.0–57.9)	**43.4**	(26.6–60.2)	**35.1**	(22.7–47.5)	**35.2**	(23.5–46.9)
card/musc/respiratory	**40.7**	(25.4–56.0)	**47.0**	(31.3–62.7)	28.2	(16.0–40.5)	24.1	(11.6–36.6)
card/musc/neurological	**40.4**	(25.1–55.8)	**45.8**	(29.9–61.6)	**36.6**	(25.1–48.2)	34.6	(23.2–46.0)
card/sens/respiratory	39.4	(26.3–52.5)	31.3	(19.9–42.7)	25.9	(14.5–37.4)	24.6	(15.4–33.7)
card/sens/neurological	38.7	(25.2–52.2)	29.6	(17.8–41.4)	34.6	(23.8–45.5)	**35.0**	(26.5–43.5)
card/respiratory/neurological	37.2	(24.9–49.6)	34.2	(24.4–44.0)	27.8	(18.5–37.1)	23.5	(16.6–30.5)
musc/sens/respiratory	31.5	(14.1–49.0)	36.8	(18.6–55.0)	32.6	(20.3–44.9)	30.7	(18.3–43.2)
musc/sens/neurological	30.6	(12.5–48.7)	34.8	(16.1–53.4)	**40.5**	(29.0–52.0)	**40.4**	(29.2–51.6)
musc/respiratory/neurological	29.0	(11.5–46.5)	39.2	(22.1–56.4)	34.2	(23.1–45.3)	29.8	(18.0–41.7)
sens/respiratory/neurological	27.0	(13.3–40.6)	20.7	(9.5–31.8)	32.2	(21.7–42.8)	30.3	(22.0–38.7)

^a^ All analyses adjusted for sex, level of education, marital status and living situation.

^b^ Models also adjusted for other disease domains

**Table 5:** Population attributable risk for instrumental activities of daily living disability by disease cluster after five years of follow up, stratified by age and sex^a^^b^.

	Age 65–74				Age 75+			
	Males		Females		Males		Females	
	PAR	95% CI	PAR	95% CI	PAR	95% CI	PAR	95% CI
***1 domain***								
cardiometabolic	**12.7**	(6.4–18.9)	12.0	(6.5–17.5)	5.6	(1.4–9.8)		
musculoskeletal			**17.2**	(5.3–29.2)			**8.8**	(0.8–16.8)
sensory	8.7	(2.5–15.0)			**7.7**	(1.9–13.4)	6.2	(0.9–11.4)
respiratory	7.3	(3.3–11.3)	5.1	(1.8–8.4)	3.1	(0.4–5.8)		
neurological	5.0	(2.0–8.0)	3.0	(0.6–5.4)	6.9	(3.9–10.0)	4.7	(2.6–6.8)
***2 domains***								
card/musc	**20.8**	(11.0–30.6)	**28.1**	(16.2–40.0)	9.5	(1.7–17.4)	11.3	(2.9–19.6)
card/sens	**21.0**	(13.0–29.0)	13.9	(6.6–21.2)	**13.3**	(6.4–20.3)	8.7	(2.6–14.7)
card/respiratory	19.7	(12.7–26.6)	16.9	(10.8–23.0)	8.8	(3.8–13.7)		
card/neurological	17.6	(10.8–24.3)	14.9	(9.1–20.7)	12.7	(7.4–17.9)	7.2	(3.2–11.3)
musc/sens	17.0	(6.6–27.4)	19.0	(6.4–31.6)	11.6	(3.1–20.2)	**15.0**	(5.7–24.3)
musc/respiratory	15.7	(6.1–25.3)	**21.9**	(10.0–33.8)			10.0	(1.8–18.1)
musc/neurological	13.6	(3.9–23.3)	**20.0**	(8.1–32.0)	10.9	(3.4–18.5)	**13.7**	(5.2–22.1)
sens/respiratory	15.9	(8.8–23.0)	7.0	(0.5–13.5)	10.8	(4.7–17.0)	7.3	(1.9–12.8)
sens/neurological	**19.8**	(10.8–28.8)			**27.4**	(16.9–37.8)	**21.3**	(11.5–31.2)
respiratory/neurological	17.9	(11.4–24.5)	11.7	(6.0–17.3)	**19.8**	(12.6–27.0)	12.2	(6.8–17.5)
***3 domains***								
card/musc/sens	**38.0**	(26.4–49.6)	**38.0**	(24.5. 51.6)	31.3	(17.6–45.0)	**31.9**	(17.7–46.1)
card/musc/respiratory	**36.3**	(25.1–47.4)	**40.7**	(27.9–53.8)	24.2	(10.9–37.0)	24.0	(10.0–38.0)
card/musc/neurological	34.3	(22.7–45.9)	**38.9**	(25.8–51.9)	30.1	(17.3–42.9)	29.8	(16.3–43.2)
card/sens/respiratory	**36.6**	(27.2–45.9)	24.9	(15.8–34.0)	30.0	(19.1–40.8)	19.5	(8.8–30.2)
card/sens/neurological	34.3	(24.8–43.7)	22.6	(13.3–31.9)	**35.7**	(25.0–46.3)	25.5	(15.1–36.0)
card/respiratory/neurological	32.4	(23.9–40.8)	26.0	(18.3–33.7)	29.0	(20.0–38.1)	16.9	(9.0–24.9)
musc/sens/respiratory	32.5	(20.2–44.8)	31.2	(16.7–45.8)	27.4	(13.8–41.1)	**30.0**	(15.6–44.4)
musc/sens/neurological	30.1	(17.3–42.9)	29.0	(14.0–44.1)	**33.4**	(20.1–46.6)	**35.3**	(21.5–49.0)
musc/respiratory/neurological	28.4	(16.4–40.4)	32.3	(18.5–46.2)	26.5	(13.9–39.0)	27.7	(14.3–41.1)
sens/respiratory/neurological	28.4	(19.3–37.4)	14.3	(5.2–23.4)	**32.1**	(21.9–42.4)	23.4	(13.5–33.2)

^a^ All analyses adjusted for sex, level of education, marital status and living situation.

^b^ Models also adjusted for other disease domains

The observed PAR for combinations of disease domains on ADL and IADL disability were compared to what would be expected based on additive model of summing the effect across included domains. For incident ADL disability in adults aged 65–74 years, the observed versus expected PAR values differed by 3.5% or less when examining combinations of two or three diseases. In participants ≥75 years, the observed PAR was between 7·2% and 11·1% higher for the neurological/sensory, neurological/respiratory, cardiometabolic/musculoskeletal/sensory and cardiometabolic/musculoskeletal/ neurological combinations for males and females. For incident IADL disability in 65–74 year old males and females, the observed PAR values for males were approximately 8% higher for the cardiometabolic/musculoskeletal, musculoskeletal/respiratory and musculoskeletal/neurological combinations compared to the expected PAR. For males, the observed PARs were more than 16% greater for the combinations of cardiometabolic/musculoskeletal/respiratory and cardiometabolic/musculoskeletal/neurological combinations compared to the expected PAR while in females the difference was approximately 6%. In males and females ≥75 years, the observed values were between 10% and 21% higher for the sensory/neurological, respiratory/neurological (males only), cardiometabolic/musculoskeletal/sensory, cardiometabolic/musculoskeletal/neurological, and musculoskeletal/sensory/neurological (males only) disease combinations.

## Discussion

This is one of the first population-based studies of older adults that investigated individual and population level associations between individual and combinations of chronic disease domains and its impact on the incidence of ADL and IADL disability. The large sample size allowed for stratification by age and sex revealing heterogeneity in these strata for the relationship between chronic diseases and disability.

The prevalence of the chronic disease domains was overall higher for older versus younger adults, though the order of least to most prevalent domains was the same. However, the magnitude of the association between chronic disease domains and disability differed by age. The odds of disability tended to be higher for the cardiometabolic and respiratory domains for younger versus older adults. This may reflect a survivorship bias. The cardiometabolic conditions included in this study are heart problems (history of hardening of the arteries, heart troubles or other blood diseases) and diabetes. Younger adults are less likely to die from heart disease compared to older adults and therefore may live with disability [22]. Similarly, respiratory problems including asthma, pneumonia, tuberculosis, emphysema, and bronchitis are increasingly fatal with age leaving younger individuals experiencing disability [23–25]. Sex differences for the relationship between chronic disease domains and disability were also observed. The musculoskeletal domain tended to be associated with disability in females but not in males which is consistent with the literature showing that females tend to have more limitations due to arthritis, the most prevalent condition in this domain, compared to males [26]. For all age and sex strata, the association of sensory disorders with disability was relatively modest compared to previous literature [27,28]. However, this may reflect the inclusion of level of education, marital status, and living situation in our models as well as the measurement of incident disability after five years compared to the cross-sectional analyses conducted in previous studies.

The PAR analyses combines both the prevalence and the odds of disability to determine the overall impact of each disease domain on disability at the population level. PAR estimates varied by age. In younger adults, disease domains with relatively high prevalence and a modest association with disability such as the cardiometabolic domain and the musculoskeletal domain were the most significant drivers of population level disability with PAR values of between 12 and 23%. In older adults, the PAR across different disease domains were more homogenous compared to younger adults ranging from 4·8 to 11·2% in the ADL sample and 3·1 to 8·8% in the IADL sample though the more prevalent disease domains still tended to have the greatest PAR estimates. In general, the respiratory conditions and cardiometabolic conditions appear to be the least important reflecting the lower prevalence of these domains compared to the cardiometabolic and musculoskeletal domains. In younger adults, the ranking of the PAR for the ADL sample and IADL sample were similar, indicating that the same disease domains can be targeted regardless of if the goal is to reduce ADL or IADL disability. However, the PAR tends to be higher in the ADL sample reflecting the higher prevalence of every disease domain in the ADL sample compared to the IADL sample.

The analyses of chronic conditions in two or three domains revealed that the combinations of disease clusters with the highest PARs were composed of the individual domains with the highest PARs. Across combinations, the PARs for incident ADL were within approximately 10% of what would be expected by adding up the PARs for the individual chronic disease domains for all age and sex strata. This suggests that having multiple chronic disease domains does not have an increased or synergistic effect on disability risk at the population level. For incident IADL, the PARs were up to 20% higher for some of the combinations of three diseases for certain age and sex strata suggesting there may be a synergistic effect. At the individual level, positive and negative interactions between chronic conditions on disability risk have been found and seem dependent on which conditions are investigated [29].

Strengths of this study include the longitudinal data which allows for the causal relationship between chronic disease domains and disability to be determined. The PAR estimates for single disease domains in this paper are smaller than those previously found using cross-sectional data from CSHA [11]. This may be reflective of reverse causation in studies using cross-sectional data, the exclusion of individuals with disability at baseline which removed numerous individuals with chronic conditions, or the combinations of disease domains. Though the large sample size available for this analyses allowed for the PAR of combinations of chronic disease domains to be assessed, it was not sufficiently large enough to allow all individual combinations of chronic diseases to be investigated and limited the number of clusters that could be included. However, the available sample size did allow for the PAR to be determined for each age and sex strata which revealed heterogeneity in the relative importance of different chronic disease domains across the strata.

This study has implications for public health initiatives. Managing less prevalent conditions that are strongly associated with disability will continue to be important on the individual level. However, the results of this study emphasize that from a public health perspective, highly prevalent conditions with more modest associations with disability may have an overall greater impact on reducing disability at the population level than interventions which target low prevalence conditions with large associations with disability. This information is complementary to studies such as the Global Burden of Disease which highlight what risk factors can be addressed to reduce the burden of individual diseases [30].

## Conclusions

The chronic disease domains which most strongly predicted incident ADLs and IADLs such as neurological conditions did not account for the greatest amount of disability at the population level measured through PARs. Instead, the most prevalent chronic conditions domains were the conditions with the highest values for PAR, even if the individual risk association with disability was lower. This suggests that public health interventions designed to target highly prevalent conditions may have a greater impact on reducing disability than focusing on less prevalent conditions with greater individual risk for disability. This may be particularly useful in the context of IADLs where the PARs were greater than expected based on an additive model suggesting a synergistic effect. Furthermore, this study highlights the heterogeneity between males and females and younger versus older adults which may provide important guidance for tailoring risk reduction programs.
